# A Simple Method of Curing Obesity

**Published:** 1891-09

**Authors:** 


### A Simple Method of Curing Obesity.

In a French journal is announced the discovery of a means, as simple as it is strange, for curing obesity which is attributed to a medical officer in the army. Thanks to this means, a colonel who was threatened to be obliged to retire from the army, as he was so heavy that it required two men to lift him into the saddle, became thin in a few weeks, and to such an extent that he had to take means to recover, in a measure, what he had lost. It was to his doctor that he was indebted to have become a general. The means consisted simply in never eating more than one dish at each meal, no matter what that dish may be, and a person may consume as much as the stomach may bear, and satisfy the appetite without the least reserve. Nevertheless, nothing but the one dish should be taken; no condiments, nor soups, nor supplementary desserts should be allowed. This system was recommended by the author to a lady who was slightly obese, and who put it into practice with the best results. The lady observed that she suffered no inconvenience whatever from this diet, and the result obtained by the medical officer may be well understood, as she found by her own experience that the partaking of one dish, whether it be meat, fish, or vegetables, brought on a sense of satiety much sooner than if she had partaken of a variety of dishes, whence the effect of relative abstinence.—*Mass. Med. Journal.*

"ASEPSIS and antisepsis form the rock upon which the edifice of modern surgery is founded, and in virtue of which it has registered triumphs in the treatment of diseased conditions of the human body formerly unattainable."—W. D. MILLER, M.D., D.D.S., Berlin.

